# Chemical Composition, Antimicrobial and Antioxidant Activities of the Flower Volatile Oils of *Fagopyrum esculentum*, *Fagopyrum tataricum* and *Fagopyrum Cymosum*

**DOI:** 10.3390/molecules23010182

**Published:** 2018-01-22

**Authors:** Jianglin Zhao, Lan Jiang, Xiaohui Tang, Lianxin Peng, Xing Li, Gang Zhao, Lingyun Zhong

**Affiliations:** 1 College of Pharmacy and Biological Engineering, Chengdu University, Chengdu 610106, Sichuan, China; jlzhao@cdu.edu.cn (J.Z.); jl25258@163.com (L.J.); tangxiaohui@mail.cdu.edu.cn (X.T.); bsmpmcereal@163.com (L.P.); lixcdu2017@163.com (X.L.); 2 Key Laboratory of Coarse Cereals Processing, Ministry of Agriculture, Chengdu 610106, Sichuan, China; ccpczhaogang@163.com

**Keywords:** volatile oil, *Fagopyrum esculentum*, *Fagopyrum tataricum*, *Fagopyrum cymosum*, antimicrobial activity, antioxidant activity

## Abstract

The purpose of this study was to investigate the chemical composition and biological activity of the volatile oils (VOs) from the flowers of three buckwheat species, *Fagopyrum esculentum*, *Fagopyrum tataricum* and *Fagopyrum cymosum*. The VOs were obtained from the fresh buckwheat flowers by hydrodistillation, and were analyzed for their chemical composition by gas chromatography-mass spectrometry (GC-MS). Nonanoic acid (7.58%), (*E*)-3-hexen-1-ol (6.52%), and benzothiazole (5.08%) were the major constituents among the 28 identified components which accounted for 92.89% of the total oil of *F. esculentum*. 2-Pentadecanone (18.61%), eugenol (17.18%), 1,2-benzenedicarboxylic acid, bis(2-methylpropyl) ester (13.19%), and (*E*,*E*)-farnesylacetone (7.15%) were the major compounds among the 14 identified components which accounted for 88.48% of the total oil of *F. tataricum*. Eugenol (12.22%), (*E*)-3-hexen-1-yl acetate (8.03%), linalool oxide (7.47%), 1-hexanol (7.07%), and benzothiazole (6.72%) were the main compounds of the 20 identified components which accounted for 90.23% of the total oil of *F. cymosum*. The three VOs were screened to have broad spectrum antibacterial activity with minimum inhibitory concentration (MIC) values ranged from 100.0 μg/mL to 800.0 μg/mL against the tested bacteria, and their median inhibitory concentration (IC_50_) values were from 68.32 μg/mL to 452.32 μg/mL. *Xanthomonas vesicatoria* was the most sensitive bacterium. Moreover, the flower VOs of *F. esculentum*, *F. tataricum* and *F. cymosum* also exhibited noteworthy antioxidant capacity with the IC_50_ value of 354.15 μg/mL, 210.63 μg/mL, and 264.92 μg/mL for the 1,1-diphenyl-2-picrylhydrazyl (DPPH) free radical scavenging assay, and the value of 242.06 μg/mL, 184.13 μg/mL, and 206.11 μg/mL respectively for the β-carotene-linoleic bleaching test. These results suggested the volatile oils of buckwheat flowers could be potential resource of natural antimicrobial and antioxidant agents.

## 1. Introduction

In recent years, increasing attention has been paid to the exploration of naturally-occurring antimicrobials and antioxidants as the growing consumer demand for agricultural byproducts free from synthetic chemical additives [1,2]. Among all natural products, essential oils (volatile components mainly include hydrocarbons, alcohols, aldehydes, esters, ketones, acids, phenols and terpenoids) originated from medicinal plants have been demonstrated to have notable antibacterial, antifungal, antiviral, antioxidant and insecticidal activities [3,4,5]. They are more beneficial for human health and environment safety, and attracted many researchers’ interests. A large number of volatile oils (VOs) have been widely used in agriculture, medicine, food and cosmetic industry [6,7,8,9].

Buckwheat belongs to the family of Polygonaceae. *Fagopyrum esculentum* (Common buckwheat) has been a crop of secondary importance in many countries, and the main producers are the Russian Federation, China, Ukraine and Kazakhstan [10,11]. *Fagopyrum tataricum* (Tartary buckwheat) is a popular edible and medicinal coarse cereal, mainly cultivated in the southwest of China, northern India, Bhutan and Nepal [12,13]. Both *F. esculentum* and *F. tataricum* are rich in proteins, amino acids, dietary fiber, vitamins and trace elements. Nowadays, there are a lot of buckwheat-based food products available on the market such as buckwheat flour, noodles, bread, tea, vinegar and sprouts [14,15,16]. *Fagopyrum cymosum* is a famous medicinal plant mainly distributed in China, Nepal, India, Bhudan and Thailand. Its rhizome was regarded as folk medicine for clearing away heat and toxic materials, removing blood stasis and expelling pus, which has been widely used for the treatment of lung diseases, rheumatism, inflammation, snakebite and traumatic injuries [17,18]. The major bioactive compounds of the three buckwheat species (*F. esculentum*, *F. tataricum* and *F. cymosum*) have demonstrated to be phenolics, flavonoids (such as rutin, quercetin, kampferol, orientin, and vitexin etc.), terpenoids, steroids, d-chiro-inositol, d-fagomine, and emodin, etc. Recent studies have revealed that these bioactive compounds had notable antioxidant, hypocholesterolemic, antidiabetic, antimicrobial, and antitumor activities, and were beneficial for human health [19,20].

To the best of our knowledge, there were no previous reports about the chemical composition, antibacterial and antioxidant activities of the volatile oils from the flowers of *F. esculentum*, *F. tataricum* and *F. cymosum*, besides a few reports have investigated the aroma volatiles of buckwheat grains and buckwheat teas, so far [10,14,21,22]. The aim of present study was to determine the aroma components of the volatile oils from the fresh flowers of these three buckwheat species, as well as to evaluate their antimicrobial and antioxidant activities for their potential applications in agriculture or the food industry. 

## 2. Results and Discussion

### 2.1. Chemical Compositions Analysis of Volatile Oils

By hydrodistillation, the volatile oil yield (*v*/*w*) of the fresh flowers obtained from *Fagopyrum esculentum*, *Fagopyrum tataricum* and *Fagopyrum cymosum* was determined as 0.28%, 0.43%, and 0.31%, respectively (Table 1).

The chemical composition of the three buckwheat flower volatile oils (VOs) was analyzed by gas chromatography-mass spectrometry. On the whole, the VOs were abundant in ketones, alcohols, esters, phenols, terpenoids, acids, aldehydes and hydrocarbons (listed in Table 2). Although, as the different varieties and geographic locations of the three buckwheat flowers used in this study, it could significantly affected the chemical composition and amount of these three VOs. For the flower volatile oil of *F. esculentum*, about twenty-eight compounds were identified, which accounted for 92.89% of the total oil. They were mainly composed of acids (24.05%), hydrocarbons (15.05%), aldehydes (14.96%) and esters (10.54%). The major components in the oil were nonanoic acid (7.58%), (*E*)-3-hexen-1-ol (6.52%), benzothiazole (5.08%), 6,10,14-trimethyl-2-pentadecanone (5.06%), and benzene-acetaldehyde (4.54%). A total of fourteen aroma constituents were determined, accounting for 88.48% of the total oil of *F. tataricum* flowers. The ketones, phenols and esters constituted the dominant portions which accounted for 25.76%, 20.98% and 16.40%, respectively. Of them, the main components were 2-pentadecanone (18.61%), eugenol (17.18%), 1,2-benzenedicarboxylic acid, bis(2-methylpropyl) ester (13.19%), (*E*,*E*)-farnesylacetone (7.15%), and heneicosane (5.28%). For the volatile oil of *F. cymosum* flowers, at least twenty compounds were identified, which accounted for 90.23% of the total oil. They were mainly composed of alcohols (24.85%), ketones (13.39%), phenols (12.22%), esters (11.02) and terpenoids (9.90%). Eugenol (12.22%), (*E*)-3-hexen-1-yl acetate (8.03%), linalool oxide (7.47%), 1-hexanol (7.07%), benzothiazole (6.72%), and 1-octanol (6.71%) were the major compounds. Some volatile components such as benzeneacetaldehyde, linalool oxide, 1-hexanol, 1-octanol, phenylethyl alcohol and 5-methyl-2-furancarboxaldehyde have also been reported in the extracts of buckwheat seeds or teas [10,14,21,22,23]. 

### 2.2. Antimicrobial Activity

The antibacterial activity of the volatile oils obtained from *F. esculentum*, *F. tataricum* and *F. cymosum* flowers were evaluated against six typical bacteria (G^+^, *B. subtilis* and *S. aureus*, G^−^, *A. tumefaciens*, *E. coli*, *P. lachrymans*, and *X. vesicatoria*), which were presented in Table 3.

In general, the three buckwheat flower VOs exhibited broad spectrum antibacterial activity, and the capacity of bacterial growth inhibition was closely related with the VOs species and its treatment dosage, as well as the tested bacterial strain. The MIC values of *F. esculentum* flower VOs on tested bacteria ranged from 400 μg/mL to 800 μg/mL, and their IC_50_ values from 226.82 μg/mL to 478.42 μg/mL. For the VOs of *F. tataricum* flower, it exhibited good antibacterial activity against all tested bacteria, and the MIC values were from 200 μg/mL to 800 μg/mL. Correspondingly, their IC_50_ values were from 106.36 μg/mL to 452.32 μg/mL. The flower VOs of *F. cymosum* showed strong antibacterial activity, and the *X. vesicatoria* was the most sensitive bacterium. The MIC values were ranging from 100 μg/mL to 400 μg/mL, and their IC_50_ values from 68.32 μg/mL to 228.62 μg/mL. On the whole, the antibacterial effect of the flower VOs of *F. cymosum* was stronger than that of *F. tataricum* and *F. cymosum*, and it could be mainly attributed to the chemical composition differences of the three VOs. The two Gram-positive bacteria seemed to be more resistant to the VOs than the four Gram-negative bacteria used in this study. Both major and minor compounds (i.e., eugenol, linalool oxide, (*E*,*E*)-farnesylacetone, benzothiazole, *t*-anethole, and (-)-α-terpineol, etc.) may contribute to the antibacterial activity of the three buckwheat flower VOs.

### 2.3. Antioxidant Activity

**Figure 1:**
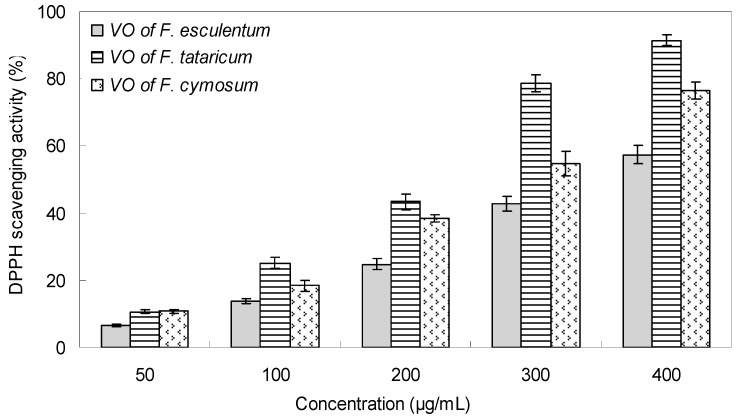
DPPH scavenging activity of the flower volatile oils of *F. esculentum*, *F. tataricum* and *F. cymosum.*

Both 1,1-diphenyl-2-picrylhydrazyl radical (DPPH^●^) scavenging and β-carotene/linoleic acid bleaching assays were applied to evaluate the antioxidant activity of the volatile oils prepared from *F. esculentum*, *F. tataricum* and *F. cymosum* flowers. The DPPH is a stable free radical which can easily experience reduction in the presence of an antioxidant [24]. Generally, the three VOs exhibited obvious activity to reduce the radical DPPH into yellow colored diphenylpicrylhydrazine, and the scavenging ability was dependent on the VOs species and concentrations. As shown in Figure 1, with concentrations ranging from 50–400 μg/mL, the DPPH radical scavenging capacity were from 6.62% to 57.36% for the flower VOs of *F. esculentum*, and from 10.62% to 91.43% for the *F. tataricum* VOs, and ranging from 10.72% to 76.48% for the *F. cymosum* VOs. Correspondingly, their IC_50_ value was determined as 354.15 μg/mL, 210.63 μg/mL, and 264.92 μg/mL, respectively (Table 4). 

**Figure 2:**
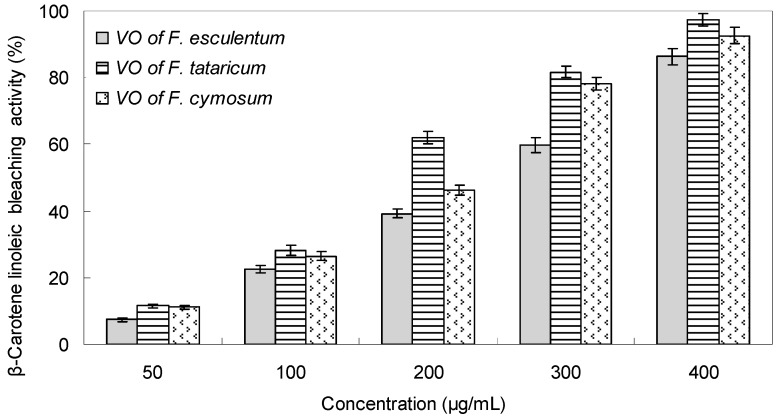
β-Carotene linoleic acid bleaching activity of the flower volatile oils of *F. esculentum*, *F. tataricum* and *F. cymosum.*

The β-carotene bleaching assay is on the basis of the lossing of yellow coloration of β-carotene due to its reaction with radicals formed by linoleic acid oxidation in an emulsion. The rate of β-carotene bleaching is slowed down by the presence of an antioxidant [25]. The antioxidant capacity of buckwheat flower VOs at different concentrations measured in β-carotene bleaching assay is displayed in Figure 2. The results indicated that the antioxidant activity of three buckwheat flower VOs increased steadily with concentrations from 50–400 μg/mL. At the concentration of 400 μg/mL, the highest β-carotene linoleic bleaching activity was 97.22% for the flower VOs of *F. tataricum*, and 92.65% for the VOs of *F. cymosum*, and that was 86.36% for the *F. esculentum* VOs. Correspondingly, their IC_50_ value was determined as 184.13 μg/mL, 206.11 μg/mL, and 242.06 μg/mL, respectively (Table 4). Generally, the flower VOs of *F. tataricum* and *F. cymosum* exhibited higher antioxidant activity than that of *F. esculentum*, and it was generally in accordance with the DPPH radical scavenging test. The main reason for the antioxidant capacity difference could be attributed to the varieties and amounts of some active constituents of the flower VOs. For example, the VOs of *F. tataricum* and *F. cymosum* are abundant in eugenol, *t*-anethole, linalool oxide and nerolidol et al., which have been proved to have notable antioxidant activity in previous reports [26,27,28,29]. 

## 3. Experimental

### 3.1. Plant Material

The fresh flowers of *Fagopyrum esculentum* and *Fagopyrum tataricum* were collected in Jintang Country (Chengdu, Sichuan Province, China) and the flowers of *Fagopyrum cymosum* were collected on Longquan Mountain (Chengdu, Sichuan Province, China) in autumn 2016, labeled and stored in sealed plastic bags at 4 °C. The taxonomical identification of the plant materials were done by Prof. Gang Zhao of Chengdu University, where the voucher specimens (CCPC-20161001, CCPC-20161002 and CCPC-20161003) of the plants were deposited.

### 3.2. Preparation of the Volatile Oils

The fresh flowers of *F. esculentum* (380.0 g), *F. tataricum* (180.0 g), and *F. cymosum* (400.0 g) were submitted to hydrodistillation in a Clevenger-type apparatus at 100 °C for 1.5–2 h, respectively. The distilled oil was extracted with diethyl ether and dried over anhydrous sodium sulfate. After filtration, the three yellow volatile oils (VOs) were obtained respectively, and then they were preserved in a sealed dark glass vial at 4 °C until required.

### 3.3. Gas Chromatography/Mass Spectrometry (GC/MS) Analysis

The chemical composition of the three buckwheat flower VOs was analyzed by the analytical GC/MS techniques. An HP-5 MS capillary column (30 m × 0.25 mm, 0.25 µm film thickness) was employed. The analysis was performed using helium (99.999%) as a carrier gas at a flow rate of 1.0 mL/min with the following temperature program: initial temperature 50 °C for 1.50 min, increase 10 °C/min up to 100 °C, 2 min at 180 °C, and then increase by 6 °C/min up to 190 °C, and increased by 20 °C/min up to 250 °C, 10 min at 250 °C. The injection volume was 1.0 µL (with a split ratio of 1:20). GC/MS analyses were carried out using an 6890N Network GC system (Agilent, CA, USA) equipped with an Agilent 5973 Network mass selective detector. Masses were scanned between 20 and 450 amu. The essential components of the VOs were identified by comparing their mass spectra with those stored in the NIST library. The retention indices (RI) of each constituent was determined relative to the retention times (RT) of a series of C_8_-C_40_ *n*-alkanes with linear interpolation on the same capillary column according to the method of Van den Dool and Kratz [30].

### 3.4. Antibacterial Activity Assay

The test bacteria included four Gram-negative (*Agrobacterium tumefaciens* ATCC 11158, *Escherichia coli* ATCC 29425, *Pseudomonas lachrymans* ATCC 11921, and *Xanthomonas vesicatoria* ATCC 11633), and two Gram-positive (*Bacillus subtilis* ATCC 11562 and *Staphylococcus aureus* ATCC 6538). They were cultivated in liquid Luria-Bertani medium overnight at 28 °C, and the diluted bacterial suspension (10^6^ cfu/mL) was ready for test. A broth dilution-colorimetric assay was employed to evaluate the antibacterial activity of the VOs [31]. In brief, the volatile oil was dissolved in acetone at an initial concentration of 20.0 mg/mL. Then it was diluted with 30% acetone to obtain serials of concentration ranging from 10.0 to 0.5 mg/mL. 10 µL of sample solutions and 90 μL of prepared bacterial suspensions were added into each well of the 96-well microplate. Streptomycin sulfate was selected as the positive control (CK^+^). After the plates were agitated to mix the contents of the wells using a plate shaker and incubated in the dark for 24 h at 28 °C, 10 µL of the exogenous reagent 3-(4,5-dimethylthiazol-2-yl)-2,5-diphenyltetrazolium bromide (MTT, 5.0 mg/mL in 0.2 mol/L, pH 7.2 phosphate-buffered saline) was added to each well, and the plates were incubated for another 4 h. The MIC value was defined as the lowest sample concentration that inhibited visible growth, as indicated by the MTT staining [32]. To confirm the MIC value, 10 µL of suspension was removed from the well and inoculated on LB medium. After incubation at 28 °C overnight, the number of surviving bacteria was calculated and certificated.

To further determine the IC_50_ value of the VOs, the microplates were centrifuged at 1500× *g* for another 15 min. Then the supernatant was aspirated, and 200 µL of dimethyl sulfoxide was added into each well. After extraction, the plate was centrifuged at 1500 *g* for another 15 min, and then 100 µL of the supernatant in each well was transferred to a corresponding well of another microplate for measurement of their light absorption values at wavelength of 510 nm. The percentage (%) of the bacterial growth inhibition was calculated as [(*A*_c_ − *A*_t_)/*A*_c_) × 100, where *A*_c_ was an average of six replicates of light absorption of the negative controls, and *A*_t_ was an average of six replicates of light absorption of the test VOs. The IC_50_ value was calculated using the linear relation between the inhibitory probability and concentration logarithm according to methods outlined by Sakuma [33].

### 3.5. DPPH Radical Scavenging Activity Assay

The DPPH radical scavenging assay of the three buckwheat flower VOs was evaluated by the method of Ono et al. [24]. In brief, 80 μL of DPPH solution (0.2 mg/mL) and 20 μL of prepared VOs solutions (0.25 to 2.0 mg/mL) were added into each well of the microplate. The mixture was shaken vigorously and kept at 37 °C for 30 min in the dark. Then, the absorbance of the solution was measured at the wavelength of 517 nm. The inhibition (%) of DPPH free radical in percent was calculated as [(*A*_control_ − *A*_sample_)/*A*_control_] × 100, where *A*_control_ is the absorbance of the control reaction containing all reagents without the test sample, and *A*_sample_ is the absorbance of the test VOs. All the tests were performed in triplicate. The 2,6-di-*tert*-butyl-4-methylphenol (BHT) was used as the positive control (CK^+^). The IC_50_ value was calculated using linear relation between the volatile oil concentration and probability of the percentage of DPPH inhibition.

### 3.6. β-Carotene-Linoleic Acid Bleaching Assay

The β-carotene-linoleic acid bleaching assay of the three volatile oils was determined according to the method of Ebrahimabadi et al. [34]. In short, 25 μL of linoleic acid and 200 mg of Tween-40 were added in the β-carotene solution (0.5 mg/mL). Then, the chloroform was removed out by the use of a rotary evaporator at 45 °C. After that, 50 mL of distilled water saturated with oxygen for 30 min at a flow rate of 100 mL/min were added, and the mixture was shaken vigorously. 90 μL of the above β-carotene-linoleic acid-Tween mixture and 10 μL of the VOs sample solutions (0.5 to 4.0 mg/mL) were added into each well. An equal amount of 30% acetone was used for the blank sample. The microplates were then kept in an incubator at 50 °C for 2 h together with BHT as the positive control (CK^+^). The absorbance of the solutions was then measured at wavelength of 460 nm. The percentage (%) of β-carotene bleaching inhibition of each sample was calculated as (*A*_β-carotene after 2 h assay_/*A*_initial β-carotene_) × 100, Where *A*_β-carotene after 2 h assay_ is the absorbance of the sample with β-carotene-linoleic acid mixture after 2 h period of incubation, and *A*_initial β-carotene_ is the absorbance of the initial mixture. The IC_50_ value calculation for β-carotene bleaching inhibition was the same as that for DPPH radical scavenging activity assay.

### 3.7. Statistical Analysis

All treatments were performed in triplicate, and the results were expressed by their mean values and the standard deviations (SD). The experimental data were submitted to analysis of variance (one-way ANOVA) to determine significant differences by PROC ANOVA of SAS version 9.2 (SAS Institute Inc., Cary, NC, USA). The term significant difference was based on *p* ≤ 0.05.

## 4. Conclusions

In conclusion, this is the first report on the chemical composition, antibacterial and antioxidant activities of the volatile oils from the fresh flowers of three buckwheat, *Fagopyrum esculentum*, *Fagopyrum tataricum* and *Fagopyrum cymosum*. Experimental data indicated that the buckwheat flower VOs were abundant in ketones, alcohols, esters, phenols, terpenoids, acids, aldehydes and hydrocarbons. They were also found to have good antibacterial activity and noteworthy antioxidant property, and that was closely related with the VOs species and its treatment dosage. These results suggested the potential application of the buckwheat flower VOs as natural antimicrobial and antioxidant agents in agriculture or food industry in the future. Although, the underlying antimicrobial and antioxidant mechanisms of the volatile oils as well as their main active constituents need to be further investigated and clarified. 

## Figures and Tables

**Table 1:** The volatile oil yield of *F. esculentum*, *F. tataricum* and *F. cymosum* flowers.

Species	Flower Biomass (g)	Volatile Oil Volume (mL)	Volatile Oil Yield (*v*/*w*, %)
*F. esculentum*	380.0	1.08	0.28
*F. tataricum*	176.0	0.75	0.43
*F. cymosum*	400.0	1.25	0.31

**Table 2:** Chemical composition of the volatile oils from the flowers of *F. esculentum*, *F. tataricum* and *F. cymosum* by GC-MS analysis.

No.	Compounds	RI ^a^	RA (%) ^b^		
			*F. esculentum*	*F. tataricum*	*F. cymosum*
1	3-Penten-2-one	1199	1.33	-	-
2	1-Pentanol	1281	-	-	1.57
3	(*Z*)-2-Penten-1-ol	1340	-	-	2.40
4	6-Methyl-5-hepten-2-one	1358	-	-	3.71
5	1-Hexanol	1367	2.40	-	7.07
6	(*E*)-3-Hexen-1-ol	1378	6.52	-	1.54
7	(*E*)-3-Hexen-1-yl acetate	1401	2.78		8.03
8	(*E*)-2-Octenal	1447	2.66	-	-
9	2-Furancarboxaldehyde	1487	-	3.25	-
10	Linalool oxide	1488	-	-	7.47
11	Pentadecane	1499	2.02	-	-
12	2-Ethyl-1-hexanol	1501	-	-	3.22
13	1-(2-Furanyl)-ethanone	1528	-	-	1.31
14	1-Octanol	1568	1.01	-	6.71
15	5-Methyl-2-furancarboxaldehyde	1595	2.76	2.50	-
16	Benzeneacetaldehyde	1669	4.54	-	-
17	Heptadecane	1699	2.99	-	-
18	(-)-α-Terpineol	1715	4.19	-	-
19	1-(3,5-Dimethyl-2-pyrazinyl)-1-ethanone	1753	-	-	4.46
20	Octadecane	1799	1.64	-	-
21	Tetradecanal	1829	2.11	-	-
22	*t*-anethole	1847	--		4.25
23	Nonadecane	1899	2.26	-	-
24	Phenylethyl alcohol	1900	-	4.60	2.34
25	1-(6,6-Dimethyl-2-methylene-3-cyclohexenyl)-buten-3-one	1959	-	-	4.36
26	(*Z*)-Jasmone	1964	-	-	5.32
27	Benzothiazole	1983	5.08	2.64	6.72
28	Eicosane	1998	3.06	-	-
29	Hexadecanal	2040	2.89	-	-
30	Isopropyl myristate	2045	4.06	-	-
31	Nerolidol	2053	-	2.18	-
32	Octanoic acid	2091	2.82	-	-
33	Heneicosane	2100	3.08	5.28	-
34	2,6-di(*t*-Butyl)-4-hydroxy-4-methyl-2,5-cyclohexadien-1-one	2117	2.70	-	-
35	2-Pentadecanone	2129	-	18.61	-
36	6,10,14-Trimethyl-2-pentadecanone	2131	5.06	-	-
37	Eugenol	2172	-	17.18	12.22
38	Nonanoic acid	2174	7.58	-	-
39	*t*-Muurolol	2206	-	-	1.43
40	Methyl hexadecanoate	2212	3.70	-	-
41	Decanoic acid	2232	3.99	-	-
42	2,4-bis(1,1-Dimethylethyl)-phenol	2249	-	3.80	-
43	Undecanoic acid	2265	3.22	-	-
44	(*E*,*E*)-Farnesylacetone	2275	-	7.15	-
45	1-Methylindole	2315	-	1.83	-
46	Dodecanoic acid	2323	2.28	-	-
47	Ethyl linoleate	2335	-	3.21	-
48	1,2-Benzenedicarboxylic acid, bis(2-methylpropyl) ester	2347	-	13.19	2.99
49	Phytol	2370	-	3.06	-
50	Hexadecanoic acid	2543	4.16	-	3.11
	Total identified		92.89	88.48	90.23
	Ketones		9.09	25.76	13.39
	Alcohols		9.93	7.66	24.85
	Esters		10.54	16.40	11.02
	Phenols		-	20.98	12.22
	Terpenoids		4.19	2.18	9.90
	Aldehydes		14.96	5.75	-

^a^ RI indicates the retention indices calculated against C_8_-C_40_ *n*-alkanes on the HP-5 MS column. ^b^ RA indicates relative amount (peak area relative to the total peak area).

**Table 3:** Antimicrobial activity of the volatile oils of three buckwheat flowers.

Test Bacterium	MIC (μg/mL)				IC_50_ (μg/mL)			
	VO of *F. esc.*	VO of *F. tat.*	VO of *F. cym.*	CK^+^	VO of *F. esc.*	VO of *F. tat.*	VO of *F. cym.*	CK^+^
*A. tumefaciens*	400	200	200	80	251.38 ± 1.76	134.06 ± 0.68	122.56 ± 0.82	45.58 ± 0.86
*E. coli*	600	400	400	60	382.46 ± 2.18	227.58 ± 1.42	213.18 ± 1.82	36.82 ± 0.62
*P. lachrymans*	400	200	200	60	248.73 ± 1.25	123.68 ± 1.26	116.28 ± 1.04	32.52 ± 0.45
*X. vesicatoria*	400	200	100	60	226.82 ± 0.83	106.36 ± 0.88	68.32 ± 0.62	31.36 ± 0.76
*B. subtilis*	800	600	400	100	436.14 ± 1.28	322.76 ± 2.38	216.36 ± 2.08	58.62 ± 1.27
*S. aureus*	800	800	400	200	478.42 ± 3.58	452.32 ± 2.16	228.62 ± 1.78	106.56 ± 1.38

The positive control (CK+) for bacteria was streptomycin sulfate. Values represent mean ± standard deviation (*n* = 3).

**Table 4:** Antioxidant activity of the volatile oils of three buckwheat flowers.

Sample	DPPH Scavenging Activity IC_50_ (μg/mL)	β-Carotene-Linoleic Bleaching Assay IC_50_ (μg/mL)
VO of *F. esculentum*	354.15 ± 3.82	242.06 ± 2.48
VO of *F. tataricum*	210.63 ± 2.68	184.13 ± 2.06
VO of *F. cymosum*	264.92 ± 1.84	206.11 ± 1.72
CK^+^	37.86 ± 0.78	25.32 ± 0.68

The positive control (CK^+^) for both DPPH scavenging and β-carotene-linoleic bleaching assays was BHT. Values represent mean ± standard deviation (*n* = 3).
